# Control of Viremia and Prevention of AIDS following Immunotherapy of SIV-Infected Macaques with Peptide-Pulsed Blood

**DOI:** 10.1371/journal.ppat.1000055

**Published:** 2008-05-02

**Authors:** Robert De Rose, Caroline S. Fernandez, Miranda Z. Smith, C. Jane Batten, Sheilajen Alcântara, Vivienne Peut, Erik Rollman, Liyen Loh, Rosemarie D. Mason, Kim Wilson, Matthew G. Law, Amanda J. Handley, Stephen J. Kent

**Affiliations:** 1 Department of Microbiology and Immunology, University of Melbourne, Parkville, Victoria, Australia; 2 National Serology Reference Laboratory, Fitzroy, Victoria, Australia; 3 National Centre for HIV Epidemiology and Clinical Research, University of New South Wales, Sydney, New South Wales, Australia; 4 Opal Therapeutics Pty Ltd, Melbourne, Victoria, Australia; National Institutes of Health-NIAID, United States of America

## Abstract

Effective immunotherapies for HIV are needed. Drug therapies are life-long with significant toxicities. Dendritic-cell based immunotherapy approaches are promising but impractical for widespread use. A simple immunotherapy, reinfusing fresh autologous blood cells exposed to overlapping SIV peptides for 1 hour ex vivo, was assessed for the control of SIV_mac251_ replication in 36 pigtail macaques. An initial set of four immunizations was administered under antiretroviral cover and a booster set of three immunizations administered 6 months later. Vaccinated animals were randomized to receive Gag peptides alone or peptides spanning all nine SIV proteins. High-level, SIV-specific CD4 and CD8 T-cell immunity was induced following immunization, both during antiretroviral cover and without. Virus levels were durably ∼10-fold lower for 1 year in immunized animals compared to controls, and a significant delay in AIDS-related mortality resulted. Broader immunity resulted following immunizations with peptides spanning all nine SIV proteins, but the responses to Gag were weaker in comparison to animals only immunized with Gag. No difference in viral outcome occurred in animals immunized with all SIV proteins compared to animals immunized against Gag alone. Peptide-pulsed blood cells are an immunogenic and effective immunotherapy in SIV-infected macaques. Our results suggest Gag alone is an effective antigen for T-cell immunotherapy. Fresh blood cells pulsed with overlapping Gag peptides is proceeding into trials in HIV-infected humans.

## Author Summary

Effective immunotherapies for HIV are needed. We assessed a simple technique, reinfusion of fresh blood cells incubating with overlapping SIV peptides (Overlapping Peptide-pulsed Autologous ceLls, OPAL), in 36 randomly allocated SIV-infected monkeys. We analyzed this therapy for the stimulation of immunity, control of virus levels, and prevention of AIDS. The OPAL immunotherapy was safe and stimulated remarkable levels of T-cell immunity. Levels of virus in vaccinated monkeys were 10-fold lower than in controls, and this was durable for over 1 year after the initial vaccinations. The immunotherapy resulted in fewer deaths from AIDS. We conclude this is a promising immunotherapy technique. Trials in HIV-infected humans of OPAL therapy are planned.

## Introduction

Several attempts at immunotherapy of HIV using more conventional vaccines have thus far been poorly immunogenic and weakly efficacious in human trials [1],[2],[3],[4]. The use of professional antigen-presenting cells such as dendritic cells to deliver HIV immunotherapies has shown strong immunogenicity efficacy in macaques and pilot humans studies but is limited to highly specialized facilities [5],[6],[7]. A simple intermittent immunotherapy that reduces the need for long-term antiretroviral therapy (ART) would be a quantum advance in treating HIV.

We recently reported the robust T-cell immunogenicity of treating unfractionated whole blood or peripheral blood mononuclear cells (PBMC) with overlapping peptides of SIV, HIV-1 or hepatitis C virus in outbred pigtail monkeys [8],[9]. We termed this simple immunotherapy OPAL (**O**verlapping **P**eptide-pulsed **A**utologous ce**L**ls). This technique is attractive since there is no prolonged *ex vivo* culture of antigen-presenting cells, robust CD4 and CD8 T-cell responses to both structural and regulatory proteins can be induced, and peptide antigens are simple to manufacture to high purity. This study assessed whether OPAL vaccination improves the outcome of SIV-infected monkeys.

Considerable debate exists regarding the most effective antigens to target for T-cell based therapeutic HIV vaccination. It has been widely believed that broader immunity to multiple proteins would be more efficacious [10],[11]. In contrast, recent studies highlight the effectiveness of Gag-specific T cell immunity in comparison to T cell immunity to other antigens. We therefore also assessed whether narrower responses induced only to SIV Gag are as effective as more broadly targeting all 9 SIV proteins.

## Materials and Methods

### Animals

Juvenile pigtail macaques (*Macaca nemestrina*) free from Simian retrovirus type D were studied in protocols approved by institutional animal ethics committees and cared for in accordance with Australian National Health and Medical Research Council guidelines. All pigtail macaques were typed for MHC class I alleles by reference strand mediated conformational analysis and the presence of Mane-A*10 confirmed by sequence specific primer PCR as described [12],[13]. 36 macaques were injected intravenously with 40 tissue culture infectious doses of SIV_mac251_ (kindly provided by R. Pal, Advanced Biosciences, Kensington, MD) as described previously [14],[15] and randomized into 3 groups of 12 animals (OPAL-Gag, OPAL-All, Controls) 3 weeks later. Randomization was stratified for peak SIV viral load at week 2, weight, gender and the MHC I gene Mane-A*10 (which is known to enhance immune control of SIV) [15]. Animals received subcutaneous injections of dual anti-retroviral therapy with tenofovir and emtricitibine (kindly donated by Gilead, Foster City, CA; both 30 mg/kg/animal) for 7 weeks from week 3: daily from weeks 3–5 post-infection and three times per week from weeks 6–10. This dual ART controls viremia in the majority of SIV-infected macaques [16],[17],[18],[19],[20].

### Immunizations

Two animal groups (OPAL-Gag and OPAL-All) were immunized with OPAL immunotherapy using PBMC as previously described [8]. Briefly, peripheral blood mononuclear cells (PBMC) were isolated over Ficoll-paque from 18 ml of blood (anticoagulated with Na^+^-Heparain). All isolated PBMC (on average 24 million cells) were suspended in 0.5 ml of normal saline to which either a pool of 125 SIV_mac239_ Gag peptides or 823 peptides spanning all SIV_mac239_ proteins (Gag, Pol, Env, Nef, Vif, Tat, Rev, Vpr, Vpx) were added at 10 µg/ml of each peptide within the pool. Peptides were 15mers overlapping by 11 amino acids at >80% purity kindly provided by the NIH AIDS reagent repository program (catalog numbers 6204, 6443, 6883, 6448-50, 6407, 8762, 6205). To pool the peptides, each 1 mg vial of lyophilised 15mer peptide was solubilized in 10–50 µl of pure DMSO and added together. The concentration of the SIV Gag and All peptide pools was 629 and 72 µg/ml/peptide respectively, although each peptide was pulsed onto cells at 10 µg/ml regardless of vaccine type. The peptide-pulsed PBMC were held for 1 hr in a 37°C waterbath, gently vortexed every 15 minutes and then, without washing, reinfused IV into the autologous animal. Peptide concentrations and timing of incubation were adapted from effective stimulation of T cell responses *in vitro*. Control macaques did not receive vaccine treatment. This was done since (a) we had not previously observed any significant VL changes with non HIV/SIV peptide sets ([8],[9] and unpublished data), (b) reinfusion of blood cells pulsed with irrelevant sets of peptides would result in some level of immune activation and drive higher viral loads in controls, artificially magnifying any reductions in the active treatment groups, (c) reinfusion of control peptide pulsed cells might have obscured any unexpected safety problems of the procedure.

### Immunology assays

SIV-specific CD4 and CD8 T-cell immune responses were analysed by expression of intracellular IFNγ as previously described [21]. Briefly, 200 µl whole blood was incubated at 37°C with 1 µg/ml/peptide overlapping 15mer SIV peptide pools (described above) or DMSO alone and the co-stimulatory antibodies anti-CD28 and anti-CD49d (BD Biosciences/Pharmingen San Diego CA) and Brefeldin A (10 µg/ml, Sigma) for 6 h. Anti-CD3-PE, anti-CD4-FITC and anti-CD8-PerCP (BD, clones SP34, M-T477 and SK1 respectively) antibodies were added for 30 min. Red blood cells were lysed (FACS lysing solution, BD) and the remaining leukocytes permeabilized (FACS Permeabilizing Solution 2, BD) and incubated with anti-human IFNγ-APC antibody (BD, clone B27) prior to fixation and acquisition (LSRII, BD). Acquisition data were analyzed using Flowjo version 6.3.2 (Tree Star, Ashland, OR). The percentage of antigen-specific gated lymphocytes expressing IFNγ was assessed in both CD3^+^CD4^+^ and CD3^+^CD8^+^ lymphocyte subsets. Responses to the immunodominant SIV Gag CD8 T-cell epitope KP9 in Mane-A*10+ animals were assessed by a Mane-A*10/KP9 tetramer as described [13]. Total peripheral CD4 T-cells were measured as a proportion of lymphocytes by flow cytometry on fresh blood.

### Virology assays

Plasma SIV RNA was quantitated by real time PCR on 140 µl of plasma at the University of Melbourne (lower limit of quantitation 3.1 log_10_ copies/ml) at all time-points using a TaqMan probe as previously described [21],[22] and, to validate these results with a more sensitive assay, on pelleted virions from 1.0 mL of plasma at the National Cancer Institute (lower limit of quantitation 1.5 log_10_ copies/ml) as previously described [23].

### Endpoints/statistical analyses

The primary endpoint was the reduction in plasma SIV RNA in OPAL-immunized animals compared to controls by time-weighted area-under-the-curve (TWAUC) for 10 weeks following withdrawal of ART (i.e. samples from weeks 12 to 20). This summary statistical approach is recommended for studies such as these involving serial measurements [24]. We compared both active treatment groups (OPAL-Gag and OPAL-All) to controls separately and together. The primary analysis was restricted to animals that controlled viremia on the ART at week 10 (VL<3.1 log_10_ copies/ml), since control of VL is an important predictor of the ability of animals to respond to immunotherapies [8],[25]. A pre-planned secondary virologic endpoint was studying all live animals adjusting for both VL at the end of ART (week 10) and Mane-A*10 status. Group comparisons used two-sample t-tests for continuous data, and Fisher's exact test for binary data. Survival analyses utilised Cox-regression analyses.

### Power calculation

Prior to initiating the study, we estimated the standard deviation of the return of VL after treatment interruption would be approximately 0.8 log_10_ copies of SIV RNA/mL plasma [5],[16],[17],[18],[19],[20]. In this intensive study we estimated that 2 of the 12 monkeys within a group may have confounding problems such as incomplete response to ART or death from acute SIV infection. A 10 control vs 10 active treatment comparison yields 80% power (p = 0.05) to detect a 1.0 log_10_ difference in TWAUC VL over the first 10 weeks. An estimated comparison of 10 control vs all 20 actively treated animals (OPAL-Gag plus OPAL-All) gave 80% power to detect differences of 0.87 log_10_ copies/ml VL reduction.

### Study conduct

This study was conducted according to a pre-written protocol using Good Laboratory Practice Standards from the Australian Therapeutic Goods Administration as a guide. Protocol deviations were minor and did not affect the results of the study. Partial data audits during the study did not raise any concerns about the study conduct.

## Results

### OPAL Vaccinations

OPAL immunotherapy was studied in SIV-infected pigtail macaques receiving ART. Pigtail macaques have at least an equivalently pathogenic course of SIV infection as alternate rhesus macaque models [14],[26]. Thirty-six macaques were infected with SIV_mac251_ and 3 weeks later treatment with the antiretrovirals tenofovir and emtricitabine for 7 weeks was initiated. The animals were randomly allocated to 3 groups stratified by peak plasma SIV viral load (VL), Mane-A*10 status (an MHC class I gene that improves VL in SIV-infected pigtail macaques [15]), weight and gender. Macaques were immunized 4 times under the cover of antiretroviral therapy (weeks 4, 6, 8, 10) with autologous fresh PBMC mixed for 1 hour *ex vivo* with 10 µg/ml/peptide of either 125 overlapping SIV Gag 15mer peptides only (OPAL-Gag), 823 SIV 15mer peptides spanning all 9 SIV proteins (OPAL-All) or un-immunized. The macaques were initially followed for 26 weeks after ceasing ART on week 10.

All 36 macaques became infected following SIV_mac251_ exposure and had a mean peak VL of 7.1 log_10_ copies/ml (Table S1). Prior to vaccination, 4 animals died during acute SIV infection with diarrhoea, dehydration, lethargy, anorexia and weight loss. The vaccinations were well tolerated, with no differences in mean weights, haematology parameters, or clinical observations in OPAL immunized animals compared to controls (data not shown).

### Immunogenicity

**Figure 1:**
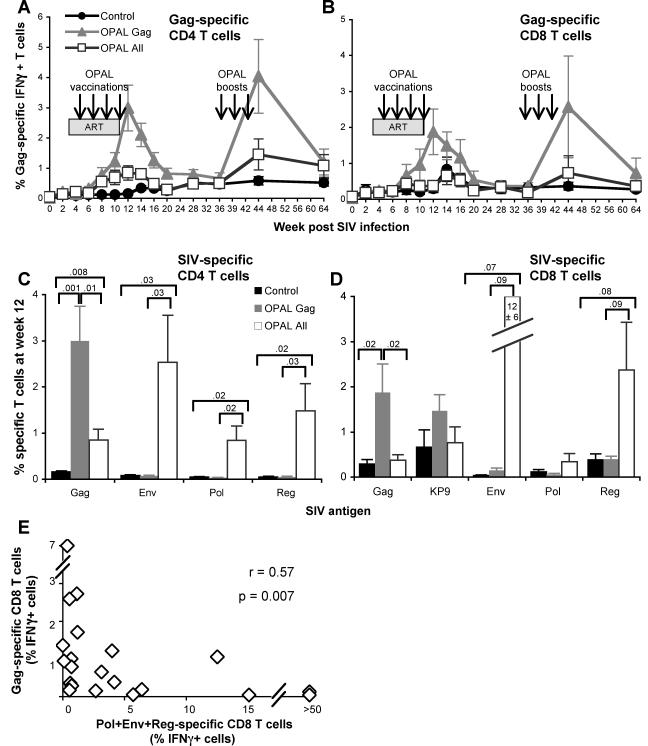
T-cell immunogenicity of OPAL vaccination. SIV Gag-specific CD4 (A) and CD8 (B) T-cells expressing IFNγ were studied over time by intracellular cytokine staining. Mean±standard error of vaccine groups compared to control unvaccinated animals (circles) is shown. The primary OPAL vaccinations of macaques (arrows, weeks 4, 6, 8 and 10 after SIV_mac251_ infection) consisted of autologous PBMC pulsed with either overlapping SIV Gag 15mer peptides (OPAL-Gag, triangles) or peptides spanning all 9 SIV proteins (OPAL-All, squares). Initial vaccinations were given under the cover of antiretroviral treatment (ART). Animals were re-boosted with OPAL immunotherapy in the same randomised groups, without ART, at weeks 39, 42 and 42. At week 12, two weeks after the last vaccination, CD4 (C) and CD8 (D) T-cell responses to pools of overlapping peptides spanning SIV Gag, Env, Pol or combined Regulatory/Accessory proteins (Nef, Tat, Rev, Vif, Vpx, Vpr [Reg]) were assessed in all animals by intracellular cytokine staining. In addition, responses to a SIV Gag CD8 T-cell epitope KP9, were assessed by a Mane-A*10/KP9 tetramer. Mean±standard error of vaccine groups is shown along with 2-sided t-test p values of <0.10. (E) SIV Gag specific CD8 T-cell responses correlated inversely with CD8 T-cell responses to the summation of non-Gag (Env+Pol+Regulatory protein) responses across all 21 live OPAL-immunized animals. The animals with >50% CD8 T-cell responses to the combined pool had total responses of 50.4% and 54.5%, primarily to Env (50.1% and 54.2% respectively). Spearman rank correlation is shown.

**Figure 2:**
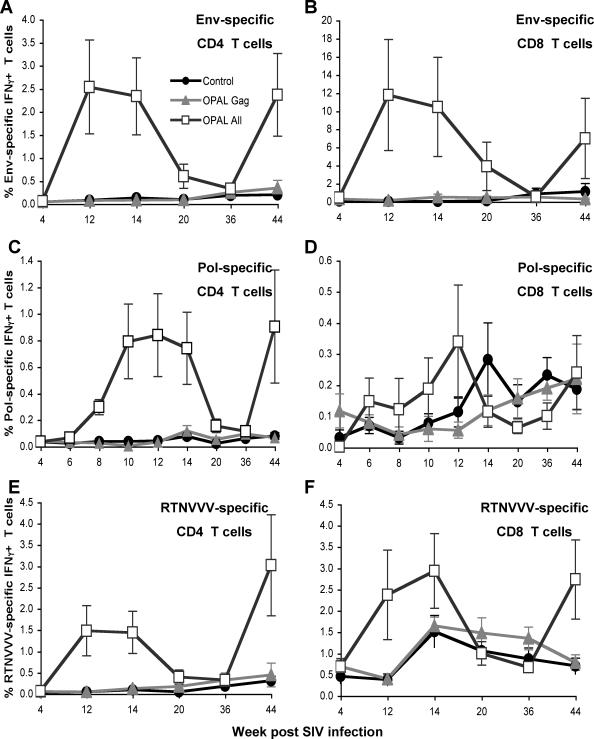
Non-Gag T cell immunogenicity of OPAL Vaccination. SIV-specific CD4 and CD8 T-cells expressing IFNγ were studied over time by intracellular cytokine staining to Env (A, B), Pol (C, D) and a pool of overlapping peptides spanning combined Regulatory/Accessory proteins (RTNVVV, E, F). Mean±standard error of vaccine groups compared to control unvaccinated animals (circles) is shown. Four initial vaccinations were given weeks 4–10 and a second set of 3 immunizations given weeks 36–42 as shown in Fig 1A.

There was striking SIV-specific CD4+ and CD8+ T-cell immunogenicity after the course of vaccination in the OPAL immunized animals. Mean Gag-specific CD4 and CD8 T-cell responses 2 weeks after the final immunization were 3.0% and 1.9% of all CD4 and CD8 T cells respectively in the OPAL-Gag group. Mean Gag-specific CD4 and CD8 T-cell responses 2 weeks after the final immunization were 0.84% and 0.37% in the OPAL-All group and 0.15% and 0.29% in controls (Fig. 1A, B). The Gag-specific T cells in the OPAL-All immunized animals, but not control or OPAL-Gag only immunized animals, also had elevated T-cell responses to all other SIV proteins. Mean Env, Pol and combined regulatory protein-specific CD4/CD8 responses were 2.5%/11.8%, 0.8%/0.3% and 1.5%/2.4% respectively in the OPAL-All group compared to ≤0.4% for all CD4/8 responses to non-Gag antigens in control and OPAL-Gag groups (Fig. 1C, D and Fig. 2). The kinetics of induction of non-Gag CD4 and CD8 T cell responses in the OPAL-All group was similar for induction of Gag-specific T cell immunity. Stronger CD8 T-cell responses to non-Gag proteins correlated with reduced CD8 T-cell responses to Gag (Fig. 1E). Thus, although a larger number of SIV proteins were recognized in the OPAL-All immunized animals, Gag responses were reduced in comparison to only immunizing with Gag peptides.

**Figure 3:**
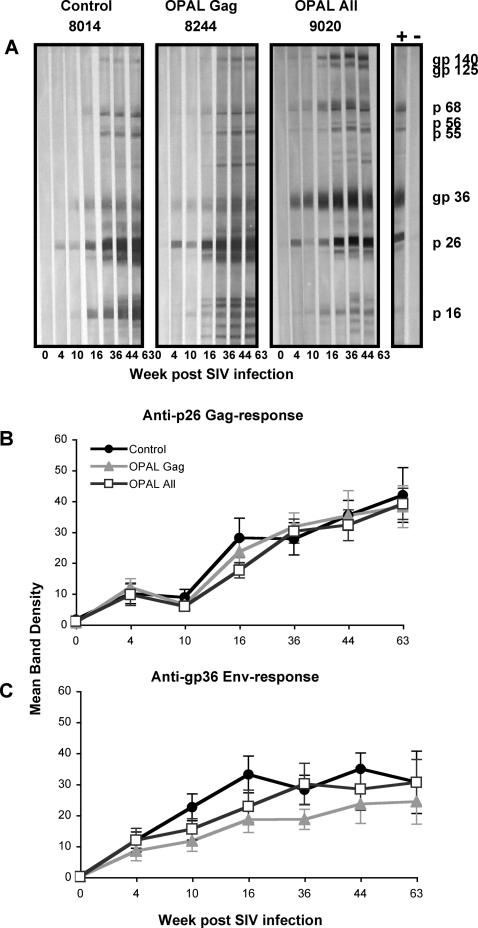
SIV-specific antibody responses. All 32 live macaques had serial measurements of SIV-specific antibodies utilizing HIV-2 Western Blot strips. (A) Representative examples of the evolution of the Western Blot profiles of a macaque within each vaccine group. A positive control sample from HIV-2 infected (+) and uninfected (−) human subjects are shown at the right of the panel. (B) Mean±standard error densitometry measurements of Gag (anti-p26) responses and (C) Env (anti-gp36) responses over time.

Although the short linear peptides were primarily used to induce T cell immunity, we also studied serial plasma samples for SIV-specific antibodies. All animals seroconverted following SIV infection, as shown by Western Blot (Fig. 3A). No significant enhancement of Gag or Env antibody responses occurred with the OPAL vaccinations (Fig. 3B, C). There was a dip in mean Gag antibody responses during the period of ART in all groups consistent with reduced viral antigen during this period. In addition to the lack of difference in mean Gag (p26) or Env (gp36) responses shown in Figure 3B and 3C, there were also no significant different antibody responses to p16, p68, gp125 and gp140 across the vaccine groups (not shown).

### Virologic outcome following initial vaccinations

The 7-week period of ART controlled VL to below 3.1 log_10_ copies/ml in 26 of the remaining 32 animals by week 10 (Table S1). The pre-defined (per-protocol) primary VL endpoint analyses was performed on animals controlling viremia on ART (26 animals). The 6 animals that failed to control viremia on ART had higher peak VLs at week 2 (mean±SD of 7.74±0.33 compared to 6.94±0.52 for animals controlling viremia on ART, p<0.001) and higher VL following ART withdrawal (5.98±0.53 vs 4.28±0.90, p<0.001). Control of VL is likely to be important in achieving optimal results from immunotherapy of infected macaques [8],[20].

**Figure 4:**
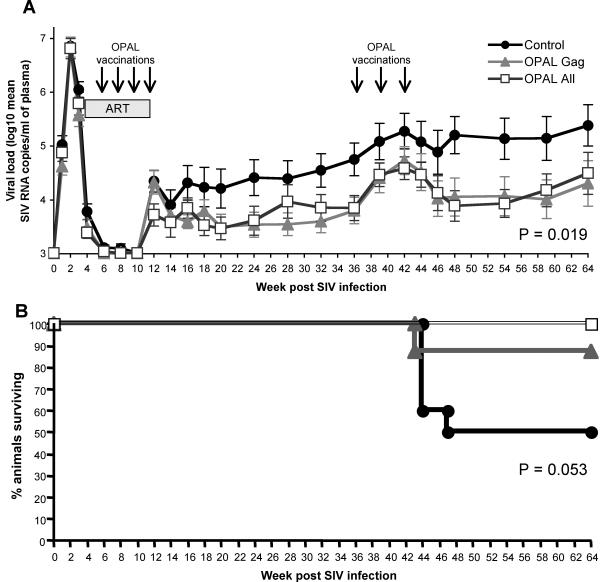
Efficacy of OPAL immunotherapy. Antiretroviral therapy (ART) was withdrawn at week 10, after the last vaccination, and (A) plasma SIV RNA followed. The 26 animals that controlled viremia on ART are illustrated with mean±standard error of vaccine groups. (B) Survival of these 26 vaccinated and controls animals is shown. P values represent the difference between controls and the combined vaccine groups (see Table 1).

The primary endpoint comparison of VL between combined OPAL-All and OPAL-Gag treatment groups in the 10 weeks after ART withdrawal was 0.5 log_10_ copies/ml lower than controls (p = 0.084, Fig 4, Table 1). Each vaccination group (OPAL-All and OPAL-Gag) had very similar reductions in VL. By 6 months after ART withdrawal, the mean difference in VL between control and OPAL-immunized groups was 0.93 log_10_ copies/ml (p = 0.028, Table 1).

**Table 1:** Statistical analyses of VL and Survival.

Animals analyzed	Comparison		n	Reduction in VL 10 weeks off ART^b^	2 sided t-test (p-value)	Reduction in VL 6 months off ART	2 sided t-test (p-value)	Reduction in VL 1 year off ART^c^	2 sided t-test (p-value)	Survival 1 year off ART (p-value)^d^
VL undetectable at week 10^a^ (n = 26)	Combined vaccine group	OPAL- Gag+All vs controls	16 vs 10	0.50 (0.73)	**0.084**	0.64 (0.93)	**0.028**	0.80 (0.98)	**0.019**	**0.053**
	Individual vaccine arms	OPAL-All vs controls	8 vs 10	0.42 (0.74)	0.136	0.61 (0.90)	0.114	0.79 (0.88)	**0.066**	NA^e^
		OPAL-Gag vs controls	8 vs 10	0.57 (0.71)	0.262	0.67 (0.95)	**0.080**	0.81 (1.07)	**0.069**	0.212
All animals, adjusted for Mane-A*10 status and VL at week 10 (n = 32)	Combined vaccine group	OPAL-Gag+All vs controls	21 vs 11	0.47 (0.54)	**0.050**	0.61 (0.66)	**0.016**	0.74 (0.66)	**0.011**	**0.020**
	Individual vaccine arms	OPAL-All vs controls	11 vs 11	0.51 (0.53)	**0.072**	0.60 (0.64)	**0.040**	0.71 (0.60)	**0.023**	**0.054**
		OPAL-Gag vs controls	10 vs 11	0.44 (0.54)	0.116	0.63 (0.69)	**0.032**	0.77 (0.72)	**0.035**	**0.022**

a 6 of the 32 animals failed to control viremia on ART.

b VL values reductions are log_10_ copies/ml compared to controls. Values shown reflect time-weighted area-under-the-curve VL between vaccinated animals and controls after coming off ART, and absolute mean reduction at the end of the period in parentheses.

c 12 animals died after week 41; the mean of the 2 last VL observations were carried forward to estimate differences in VL to week 64.

d Survival p-value reflect Cox-regression analysis.

e None of the 8 OPAL-All vaccinated animals that had VL undetectable on ART died compared to 5 of 10 controls – this comparison did not permit an estimate of significance of this comparison.

As a secondary endpoint, we also analysed all 32 remaining animals by adjusting for VL control on ART and Mane-A*10 status. There was a significant difference in VL between controls and vaccinated macaques with these analyses at both 10 and 26 weeks off ART (p = 0.050, 0.016 respectively, Table 1).

To confirm the virologic findings using a sensitive independent VL assay, frozen plasma (1 ml) from study week 32 was shipped to the National Cancer Institute (NCI) in Maryland, USA. Drs M Piatak and J Lifson kindly analysed the samples for SIV RNA blindly using an assay with a limit of quantitation of 1.5 log_10_ copies/ml (Table S1) [23]. The University of Melbourne and NCI assays were tightly correlated (r = 0.97, p<0.001) and showed an almost identical mean reduction in viremia in vaccinees compared to controls at this time (0.82 vs 0.88 log_10_ copies/ml respectively).

### Durability of OPAL immunotherapy

To further assess the durability of SIV control and prevention of disease with OPAL immunotherapy, we re-boosted all 32 animals in the same randomized groups 3 times with the identical procedure (at week 36, 39, 42) without ART cover and followed the animals for an additional 6 months. Despite the lack of ART cover, SIV-specific T cell immunity was dramatically enhanced in immunized animals 2 weeks after the last vaccination, similarly to the primary vaccination (Figs 1, 2). The T cell responses to Gag were again highest in the OPAL-Gag group with broader responses in the OPAL All group. The pattern of enhancement of T cell immunity was similar for the first and second vaccination sets (Figs 1, 2).

We again sampled plasma for viral load every 3–6 weeks. To account for the death of animals from AIDS, we used a “last observation carried forward” analysis for missing VL data. Significant viral control was maintained throughout the follow up period of just over 1 year off ART (Fig 4A, Table 1). In animals which controlled VL on ART, there was a mean 0.98 log_10_ copies/ml difference between controls and vaccinees 54 weeks after coming off ART (p = 0.019 for time-weighted analysis).

Twelve of the remaining 32 animals developed incipient AIDS and were euthanised during the extended follow up. All 6 animals that did not control viremia on ART required euthanasia. Of the 6 euthanised animals which did control viremia on ART, 5 were in the control group and one in the OPAL-Gag group. OPAL immunotherapy resulted in a survival benefit, analysing either the 26 animals that controlled viremia on ART (p = 0.053, Fig 4B, Table 1) or all 32 animals, adjusted for Mane-A*10 status and control of viremia on ART (p = 0.02, Table 1).

## Discussion

In summary, OPAL immunotherapy, either using overlapping Gag SIV peptides or peptides spanning the whole SIV proteome was highly immunogenic and resulted in significantly lower viral loads and a survival benefit compared to unvaccinated controls. The virologic efficacy in OPAL-immunized macaques was durable for 12 months after ART cessation. Our findings on OPAL immunotherapy were observed despite the virulent SIV_mac251_-pigtail model studied [14] and provide strong proof-of-principle for the promise of this immunotherapy technique.

The OPAL immunotherapy approach is simpler than many other cellular immunotherapies, particularly the use of dendritic cells. The use of DNA, CTLA-4 blockade and viral vector based approaches are also now showing some promise in macaque studies [17],[27], although such approaches have not yet been translated into human studies. This study added peptides to PBMC, however we have shown an even simpler technique, adding peptides to whole blood is also highly immunogenic, a technique that will be more widely applicable ([8] and unpublished studies).

This is one of the largest therapeutic SIV vaccine studies yet reported. Although it may have been ideal to have studied irrelevant peptide-pulsed autologous cells as an additional control group, we were concerned that this may have magnified the therapeutic effect or obscured any safety concerns. In the end, the vaccination process was both safe and effective.

How well the findings on OPAL immunotherapy translate to humans with acute HIV-1 infection will be determined by clinical trials. Virus-specific CD4 T cells are typically very weak in HIV-infected humans or SIV-infected macaques; dramatic enhancement of these cells were induced by OPAL immunotherapy and this may underlie its efficacy [28]. We measured IFNγ-producing T cells in this study since we had not developed polyfunctional ICS assays prior to initiating the study. However, recent cross-sectional polyfunctional ICS assays suggests OPAL immunotherapy can also induce T cells capable of also expressing the cytokines TNFα and IL-2, the chemokine MIP1β and the degranulation marker CD107a (unpublished data).

A ∼1.0 log_10_ reduction in VL would result in a substantial delay in progressive HIV disease in humans and allow a reasonable time period without the requirement to reintroduce ART [29] if these findings are confirmed in human trials. Both the control and vaccinated macaques were treated with ART early in this study (3 weeks after infection), which alone can be associated with a transiently improved outcome in humans [30]. None-the-less, a massive loss of CD4+ T cells in the gut occurs within 2 weeks of infection [31]. Although it may be challenging to identify humans within 3 weeks of infection, this is when HIV-1 subjects typically present with acute infection. The durable control of viremia exhibited by the vaccinated animals is interesting and consistent with other recent macaque studies [27], suggesting the need for re-immunization may not be substantial. We cannot attribute the durable control of viremia to the second set of immunizations; there was only a marginal, non-significant, increase in the difference in VL between OPAL vaccinees and controls before and after the second immunization series. Further studies are required to address the timing and benefit of ART cover during boosting immunizations with OPAL immunotherapy.

Control of viremia was similar for the OPAL-Gag and OPAL-All groups. Gag-specific CD4 and CD8 T-cell responses in OPAL-Gag animals 5.1- and 3.5-fold greater than those in the OPAL-All animals, despite an identical dose of Gag overlapping peptides. This suggests antigenic competition between peptides from Gag and the other SIV proteins. Inducing immunodominant non-Gag T-cell responses by multi-protein HIV vaccines may limit the development of Gag-specific T-cell responses [21]. A large human cohort study demonstrated Gag-specific T-cell responses were the most effective in controlling HIV viremia [32]. Useful subdominant T cell responses may be particularly susceptible to dominant non-Gag T cell responses [33],[34]. The utility, if any, of inducing T-cell responses to non-Gag proteins (i.e. excluding Gag peptides from the vaccine antigens) can be addressed in future studies of this flexible vaccine technology. Therapeutic HIV vaccines may not need to aim for maximally broad multi-protein HIV-specific immunity.

OPAL immunotherapy with Gag peptides is proceeding into initial trials in HIV-infected humans. Additional peptides can readily be added into standard consensus strains mixes to cover common strain or subtype variations between strains with this technology [35]. Additional technologies such as toggling variable amino acids peptides may provide further T cell immunogenicity with this general technology [36]. Immunotherapy with peptides delivered onto fresh blood may have potential applicability for other chronic viral diseases such as hepatitis C virus infection and some cancers such as melanoma [37].

## Supporting Information

Table S1 Viral load data
(0.08 MB PDF)
