# Supplementary material for: Control of Viremia and Prevention of AIDS following Immunotherapy of SIV-Infected Macaques with Peptide-Pulsed Blood
Source: PLoS Pathog. 2008 May 2;4(5):e1000055. doi: 10.1371/journal.ppat.1000055 (PMC2323103; doi:10.1371/journal.ppat.1000055)
Supplement: Table S1 — Viral load data (0.08 MB PDF) [file ppat.1000055.s001.pdf]

Supplementary Table 1: Viral Load

Plasma SIV RNA (log10 copies/ml) — Week of Vaccine Study

Group	Macaque ID	Mane- A*10	Gender	Weight (week 0)	0	1	2	3	4	6	8	10	12	14	16	18	20	24	28	32	32 (NCI)[b]	36	39	42	44	46	48	54	59	64	Comment
**Control**	6169	Neg	F	3.10	3.11[a]	5.48	7.14	6.33	3.61	3.26	3.11	3.11	3.94	3.11	3.33	3.11	3.11	3.66	3.44	3.11	3.04	3.76	4.12	4.09	4.23	3.70	4.21	4.49	4.35	5.89	
	8014	Pos	F	2.30	3.11	4.73	6.69	6.09	3.19	3.11	3.99	3.11	4.55	3.11	4.05	4.03	3.61	3.75	4.01	4.31	4.62	4.63	5.45	5.47	5.34	4.72	5.16	5.01	5.46	6.05	
	8252	Neg	M	3.10	3.11	4.91	7.59	5.90	4.32	3.11	3.11	3.11	5.43	4.86	5.04	5.29	5.10	5.38	4.91	5.52	5.60	5.42	6.10	6.19	5.88	5.51	5.70	5.70	5.70	5.70	
	8436	Neg	M	4.35	3.11	4.94	6.54	6.55	4.29	3.33	3.11	3.11	4.72	4.88	6.11	6.08	5.48	5.01	5.47	5.27	5.59	5.48	6.33	6.58	6.26	6.42	6.42	6.42	6.42	6.42	
	8868	Neg	F	3.35	3.11	5.34	7.38	6.23	4.04	3.11	3.11	3.11	4.72	4.37	4.51	4.53	4.83	4.90	5.00	4.74	5.18	5.21	5.70	5.70	5.56	5.63	5.63	5.63	5.63	5.63	"last observation carried forward" values[c]
	8883	Neg	M	2.25	3.11	6.06	7.25	6.74	4.21	3.54	3.11	3.11	5.24	4.90	5.27	5.25	5.84	5.88	6.11	6.37	5.68	6.52	6.34	6.79	6.97	6.88	6.88	6.88	6.88	6.88	
	9017	Pos	M	2.85	3.11	4.97	6.26	5.73	3.38	3.11	3.11	3.11	3.91	3.23	3.11	3.25	3.11	3.22	3.11	3.41	3.04	3.58	3.38	3.50	3.11	3.11	3.11	3.25	3.11	3.29	
	9019	Neg	M	3.25	3.11	4.81	7.36	6.07	3.79	3.35	3.11	3.11	4.62	5.09	5.06	5.56	5.56	5.98	5.30	5.10	4.90	5.32	5.42	5.55	5.41	5.48	5.48	5.48	5.48	5.48	last observation carried forward
	9176	Pos	M	3.80	3.11	4.21	6.30	5.23	3.46	3.11	3.11	3.11	3.94	3.40	3.11	3.11	3.11	3.24	3.11	4.01	4.11	3.60	3.74	4.77	3.60	3.28	4.71	3.40	3.23	3.34	
	9183	Pos	M	4.20	3.11	4.97	6.74	5.85	3.61	3.11	3.11	3.11	3.43	3.11	4.59	3.16	3.36	4.12	4.52	4.64	4.56	5.01	5.26	5.13	5.43	5.13	5.73	6.10	6.17	6.14	
	7992	Neg	F	3.35	3.11	5.88	8.04	7.16	5.01	5.49	5.40	**4.90**	**5.58**	**5.62**	**6.16**	**6.17**	**6.83**	**6.18**	**6.40**	**6.39**	**6.66**	**6.86**	**7.15**	**6.77**	**6.96**	**6.96**	**6.96**	**6.96**	**6.96**	**6.96**	VL>3.11 wk 10[d], last observation carried forward
	9182	Neg	M	2.45	3.11	5.96	7.26	6.92	4.79																						deceased
**Mean of group (all animals)**				3.20	3.11	5.19	7.05	6.23	3.98	3.42	3.40	3.27	4.55	4.15	4.58	4.50	4.54	4.67	4.67	4.81	4.82	5.04	5.36	5.50	5.34	5.17	5.45	5.39	5.40	5.62	
**SE**				0.20	0.00	0.16	0.16	0.16	0.16	0.21	0.22	0.16	0.21	0.29	0.33	0.37	0.40	0.34	0.35	0.32	0.34	0.33	0.36	0.33	0.38	0.41	0.34	0.38	0.40	0.37	
**Mean of group (animals with VL≤3.11 at wk 10)**					3.11	5.13	6.96	6.15	3.88	3.22	3.20	3.11	4.45	4.01	4.42	4.34	4.31	4.51	4.50	4.65	4.63	4.85	5.18	5.38	5.18	4.99	5.30	5.24	5.24	5.48	
**SE**					0.00	0.16	0.14	0.15	0.15	0.05	0.09	0.00	0.20	0.28	0.32	0.36	0.36	0.33	0.33	0.31	0.31	0.30	0.34	0.33	0.38	0.40	0.34	0.38	0.41	0.38	
**OPAL Gag**	9196	Neg	M	3.50	3.11	4.77	6.43	5.48	3.54	3.11	3.11	3.11	3.76	3.50	3.32	4.00	3.11	3.65	3.73	3.90	3.59	3.72	4.67	5.25	5.14	4.66	4.52	4.49	3.92	4.46	
	6804	Neg	M	3.20	3.11	4.57	6.73	5.40	3.76	3.11	3.11	3.11	4.97	4.16	3.68	4.37	3.66	3.11	3.18	3.47	3.54	3.69	4.80	5.36	4.81	3.88	3.85	4.21	3.98	4.45	
	8012	Neg	M	3.75	3.11	4.64	7.24	6.52	4.32	3.12	3.11	3.11	5.07	4.82	4.13	4.69	5.12	4.79	4.33	4.52	5.11	4.58	5.10	5.50	5.30	5.30	5.30	5.30	5.30	5.30	last observation carried forward
	8020	Positive	M	2.70	3.11	4.75	7.09	5.92	3.31	3.11	3.28	3.11	5.12	3.80	4.07	4.45	3.39	4.36	4.47	4.52	4.80	4.94	5.64	5.55	5.95	5.25	5.56	5.78	5.69	6.29	
	8244	Positive	M	3.15	3.11	4.00	6.00	4.76	3.11	3.11	3.11	3.11	3.83	3.72	3.67	3.72	3.80	3.88	4.16	3.88	4.00	4.56	4.87	5.01	5.11	4.49	4.67	4.25	4.68	5.34	
	8454	Positive	F	2.50	3.11	4.99	6.47	5.91	3.49	3.11	3.11	3.11	3.67	3.11	3.11	3.11	3.11	3.11	3.11	3.11	2.72	3.11	3.33	4.35	3.11	3.11	3.13	3.11	3.11	3.11	
	8673	Neg	M	4.50	3.11	4.28	6.62	5.60	3.17	3.11	3.11	3.11	4.67	3.38	3.57	3.12	3.13	3.11	3.11	3.11	2.72	3.18	3.94	3.93	3.81	3.20	3.11	3.11	3.11	3.11	
	8873	Neg	F	3.70	3.11	5.89	7.71	6.05	3.33	3.11	3.11	3.11	4.32	4.07	4.02	3.72	3.46	3.11	3.11	3.11	2.54	3.44	3.73	3.70	3.73	3.11	3.11	3.11	3.11	3.21	
	6597	Neg	M	3.50	3.11	5.18	8.04	7.15	5.33	4.59	3.99	**3.31**	**4.26**	**3.89**	**4.70**	**4.93**	**4.84**	**5.03**	**5.67**	**5.92**	**6.08**	**5.87**	**4.87**	**5.01**	**5.11**	**4.49**	**4.80**	**4.80**	**4.80**	**4.80**	VL>3.11 wk 10, last observation carried forward
	8241	Positive	M	3.35	3.11	5.22	7.30	6.97	4.60	3.93	4.14	**3.97**	**5.55**	**5.19**	**6.29**	**6.17**	**6.35**	**6.65**	**6.34**	**6.13**	**6.53**	**6.38**	**6.79**	**6.91**	**6.81**	**6.86**	**6.86**	**6.86**	**6.86**	**6.86**	VL>3.11 wk 10, last observation carried forward
	9018	Neg	F	2.15	3.11	4.40	7.32	6.46	3.36																						deceased
	9180	Neg	M	3.55	3.11	4.90	7.78	4.60	4.44																						deceased
**Mean of group (all animals)**				3.30	3.11	4.80	7.06	5.90	3.81	3.34	3.32	3.22	4.52	3.96	4.06	4.23	4.00	4.08	4.12	4.17	4.16	4.35	4.77	5.06	4.89	4.43	4.49	4.50	4.46	4.69	
**SE**				0.18	0.00	0.14	0.18	0.23	0.20	0.16	0.13	0.09	0.21	0.20	0.29	0.29	0.34	0.37	0.36	0.35	0.45	0.36	0.31	0.29	0.35	0.38	0.39	0.39	0.40	0.41	
**Mean of group (animals with VL≤3.11 at wk 10)**					3.11	4.72	6.94	5.67	3.58	3.11	3.13	3.11	4.43	3.82	3.70	3.90	3.60	3.64	3.65	3.70	3.63	3.90	4.51	4.83	4.62	4.12	4.15	4.17	4.11	4.41	
**SE**					0.00	0.16	0.18	0.20	0.15	0.00	0.02	0.00	0.22	0.19	0.13	0.21	0.24	0.23	0.21	0.21	0.34	0.25	0.27	0.26	0.34	0.33	0.35	0.36	0.36	0.42	
**Difference after ARVs ceased b/w Control & OPAL Gag group (all animals)**												0.06	0.03	0.19	0.52	0.28	0.54	0.59	0.55	0.64	0.65	0.69	0.59	0.45	0.45	0.73	0.96	0.89	0.94	0.92	
**Difference after ARVs ceased b/w Control & OPAL Gag group (animals with VL≤3.11 at wk 10)**												0.00	0.02	0.19	0.72	0.44	0.71	0.88	0.85	0.95	1.00	0.95	0.67	0.55	0.56	0.86	1.15	1.07	1.13	1.07	
**OPAL All**	1.3731	Positive	M	3.85	3.11	5.62	7.34	6.70	3.62	3.11	3.11	3.11	4.48	3.11	4.00	3.67	3.97	4.91	4.64	4.39	4.40	4.42	4.67	4.78	4.32	4.29	4.28	3.64	3.66	3.87	
	8240	Positive	F	3.40	3.11	4.29	6.73	5.36	3.11	3.11	3.11	3.11	3.11	3.11	3.11	3.11	3.11	3.11	3.11	3.11	2.38	3.11	3.62	4.41	3.78	3.11	3.11	3.51	3.33	3.19	
	8251	Neg	M	3.00	3.11	5.07	7.87	6.44	4.09	3.35	3.11	3.11	4.66	4.79	4.85	4.57	4.72	4.91	5.12	4.68	4.59	4.08	5.01	5.03	5.03	4.55	4.39	4.71	5.13	5.83	
	8680	Neg	F	3.05	3.11	4.67	6.77	5.70	3.49	3.11	3.11	3.11	3.80	3.25	3.48	3.90	3.32	3.42	5.08	4.72	5.00	4.55	5.07	4.97	4.97	4.42	4.50	4.42	5.24	5.75	
	8682	Neg	M	2.70	3.11	5.42	7.40	6.32	3.75	3.12	3.11	3.11	4.11	3.64	4.04	3.14	3.11	3.11	3.11	3.52	3.56	3.58	4.09	3.87	4.07		3.48	3.12	3.47	4.54	
	9020	Positive	M	3.15	3.11	4.29	6.21	4.61	3.17	3.11	3.11	3.11	3.11	3.11	4.07	3.11	3.11	3.11	3.11	3.13	3.04	3.11	3.94	3.94	3.93	3.11	3.11	3.30	3.44	3.11	
	9021	Positive	M	2.65	3.11	5.07	6.64	5.77	3.16	3.11	3.11	3.11	3.82	4.99	4.37	4.14	4.00	3.75	3.87	3.51	3.28	3.86	4.78	4.84	4.71	4.62	3.62	4.53	4.92	5.12	
	9175	Positive	M	3.70	3.11	5.30	6.16	6.04	3.41	3.11	3.11	3.11	3.50	3.48	3.66	3.39	3.23	3.42	4.51	4.57	4.70	4.91	5.36	5.66	5.74	5.48	5.43	5.05	5.05	5.36	
	2.3308	Neg	M	4.30	3.11	5.08	7.63	7.05	4.97	4.88	5.08	**4.38**	**4.78**	**5.34**	**5.65**	**5.88**	**5.44**	**5.86**	**5.70**	**5.81**	**5.83**	**5.78**	**6.35**	**6.40**	**5.94**	**6.17**	**6.17**	**6.17**	**6.17**	**6.17**	VL>3.11 wk 10, last observation carried forward
	8247	Neg	F	2.95	3.11	4.12	7.44	5.86	4.10	3.11	3.48	**3.69**	**5.59**	**5.61**	**5.10**	**4.83**	**4.61**	**5.48**	**5.44**	**5.37**	**5.85**	**5.47**	**5.86**	**6.08**	**5.97**	**5.97**	**5.97**	**5.97**	**5.97**	**5.97**	VL>3.11 wk 10, last observation carried forward
	8676	Neg	M	3.85	3.11	5.52	8.02	7.32	4.89	4.96	3.92	**3.88**	**5.43**	**4.99**	**5.13**	**5.45**	**5.47**	**5.46**	**5.43**	**5.66**	**5.80**	**5.55**	**6.12**	**6.26**	**6.39**	**6.33**	**6.33**	**6.33**	**6.33**	**6.33**	VL>3.11 wk 10, last observation carried forward
	8881	Neg	M	2.30	3.11	5.04	7.08	6.10	3.61																						deceased
**Mean of group (all animals)**				3.24	3.11	4.96	7.11	6.10	3.78	3.46	3.40	3.35	4.22	4.13	4.32	4.11	4.01	4.23	4.47	4.41	4.40	4.40	4.99	5.11	4.99	4.80	4.58	4.61	4.79	5.02	
**SE**				0.17	0.00	0.15	0.18	0.21	0.18	0.22	0.18	0.13	0.26	0.30	0.24	0.29	0.28	0.33	0.30	0.30	0.36	0.29	0.27	0.27	0.28	0.37	0.37	0.35	0.34	0.35	
**Mean of group (animals with VL≤3.11 at wk 10)**					3.11	4.98	6.91	5.89	3.49	3.14	3.11	3.11	3.82	3.68	3.95	3.63	3.57	3.72	4.07	3.95	3.87	3.95	4.57	4.69	4.57	4.23	3.99	4.03	4.28	4.60	
**SE**					0.00	0.16	0.19	0.21	0.11	0.03	0.00	0.00	0.20	0.27	0.19	0.19	0.21	0.27	0.31	0.25	0.33	0.23	0.22	0.21	0.24	0.32	0.28	0.26	0.31	0.39	
**Difference after ARVs ceased b/w Control & OPAL All group (all animals)**												-0.07	0.34	0.02	0.26	0.39	0.53	0.43	0.20	0.40	0.42	0.64	0.37	0.39	0.35	0.36	0.87	0.78	0.61	0.60	
**Difference after ARVs ceased b/w Control & OPAL All group (animals with VL≤3.11 at wk 10)**												0.00	0.63	0.32	0.47	0.71	0.74	0.80	0.43	0.69	0.76	0.90	0.62	0.69	0.61	0.76	1.31	1.20	0.96	0.89	
**OPAL Gag + All combined**																															
**Mean of group (all animals)**					3.11	4.88	7.08	6.00	3.80	3.40	3.36	3.29	4.36	4.05	4.19	4.16	4.00	4.16	4.30	4.29	4.29	4.38	4.89	5.09	4.94	4.62	4.54	4.56	4.63	4.87	
**SE**					0.00	0.10	0.12	0.15	0.13	0.14	0.11	0.08	0.17	0.18	0.18	0.20	0.21	0.24	0.23	0.22	0.28	0.22	0.20	0.19	0.21	0.26	0.26	0.26	0.26	0.27	
**Mean of group (animals with VL≤3.11 at wk 10)**					3.11	4.84	6.93	5.77	3.54	3.13	3.12	3.11	4.12	3.75	3.82	3.76	3.58	3.68	3.86	3.83	3.75	3.93	4.54	4.76	4.59	4.17	4.07	4.10	4.20	4.50	
**SE**					0.00	0.11	0.13	0.14	0.09	0.01	0.01	0.00	0.16	0.16	0.12	0.14	0.15	0.17	0.19	0.16	0.23	0.16	0.17	0.16	0.20	0.22	0.22	0.22	0.23	0.28	
**Difference after ARVs ceased b/w Control & OPAL Gag + All group (all animals)**												-0.01	0.19	0.10	0.38	0.34	0.54	0.51	0.37	0.51	0.53	0.66	0.48	0.42	0.40	0.55	0.92	0.83	0.77	0.75	
**Difference after ARVs ceased b/w Control & OPAL Gag + All group (animals with VL≤3.11 at wk 10)**												0.00	0.32	0.26	0.60	0.57	0.73	0.84	0.64	0.82	0.88	0.93	0.65	0.62	0.58	0.81	1.23	1.13	1.05	0.98	

[a]VL of 3.11 is the lower limit detected by University of Melbourne assay.

[b]32(NCI) represents confirmatory VL assay performed at NCI.

[c]Shaded figures represent "last observation carried forward" values for deceased animals (average of the last two available time points).

[d]Bolded figures represent animals that failed to control viremia on ARV therapy (VL > 3.11 at week 10).
